# Applying Big Data Methods to Understanding Human Behavior and Health

**DOI:** 10.3389/fncom.2018.00084

**Published:** 2018-10-16

**Authors:** Ahmed A. Moustafa, Thierno M. O. Diallo, Nicola Amoroso, Nazar Zaki, Mubashir Hassan, Hany Alashwal

**Affiliations:** ^1^School of Social Sciences and Psychology, MARCS Institute for Brain and Behaviour, Western Sydney University, Sydney, NSW, Australia; ^2^Department of Social Sciences, College of Arts and Sciences, Qatar University, Doha, Qatar; ^3^Dipartimento Interateneo di Fisica “M. Merlin, ” Università degli Studi di Bari “A. Moro, ” Bari, Italy; ^4^Istituto Nazionale di Fisica Nucleare, Sezione di Bari, Bari, Italy; ^5^College of Information Technology, United Arab Emirates University, Al-Ain, United Arab Emirates; ^6^Department of Biological Sciences, College of Natural Sciences, Kongju National University, Gongju, South Korea

**Keywords:** machine learning, deep learning, psychology, health, human behavior, big data, causality, longitudinal data

## Introduction

While many fields have benefited greatly from the collection and analysis of big data, some health fields and, to a large extent, psychology are still lagging behind (Azmak et al., 2015). Azmak et al. (2015) have shown an example (e.g., Sloan Digital Sky) on how the collection of large datasets has aided researchers to solve difficult problems in astronomy that were not possible in the past. Interestingly, the slow process of applying big data to psychology mirrors the history of development of sciences, as astronomy and other sciences are much older than experimental psychology (which emerged in the nineteenth century). This is related to the fact that while many sciences are data-driven, psychology, to a large degree, is hypothesis-driven (see below discussion on these points).

## Why big data methods have been rarely applied to psychology?

There are several reasons why psychology researchers rarely collect large datasets, and if we do, may not use big data methods for analyses. As pointed out by Cheung and Jak (2016), big data analysis is not considered a core topic in behavioral sciences. Another factor is most psychology research is theory- rather than data-drive (Qiu et al., 2018). Accordingly, most psychology researchers often collect data using a small number of variables to test their theory. Psychology students are often encouraged to have a hypothesis underlying their new experiments. However, there are often many theories that explain a certain behavioral phenomenon, and a theory-driven approach can rarely find best theories. Accordingly, here we argue that it is good to let the data speak for themselves, that is, to take a data-driven approach. However, this requires the collection of large datasets and conducting big data analyses.

Historically and up till recently, most psychological studies collect data using small number of variables (usually under 10) (for discussion see Cheung and Jak, 2016). There are, however, some exceptions including the World Values Survey, Math Garden, Kavli Human Project (Azmak et al., 2015), as well as few recent studies (Kern et al., 2014; Youyou et al., 2015). Most of these studies often analyze big data collected from social media websites, such as Facebook and Twitter. However, even with big datasets, most psychology researchers still divide the data into smaller parts for more standard statistical analyses. This is in contrast to neural (e.g., neuroimaging, EEG, and single-cell recording) that often include 100s of variables.

Further, many of the variables in psychological studies are categorical, such as male/female, lives in Urban vs. rural area, patient or control, Young vs. older, and so on. It is possible that the nature of such data have discouraged researchers from conducting complex analytical tools, as most existing deep learning and big data methods often deal with continuous variables. However, some recent efforts have shown that deep learning methods can also be applied to categorical variables. For example, Zhang et al. (2016) have used several DNNs, including Factorization Machine, supported Neural Network (FNN), and Restricted Boltzmann Machine to understand online advertisement, specifically to predict user responses in a website. While this domain is different from medical and behavioral fields, the data they have used also include categorical variables. Most of these DNNs represent categorical variables as a set of binary values. We argue that these algorithms can be applied to solve complex psychology and health problems.

## Why do we need big data analysis in psychology?

It is important to note human behavior and health issues are quite complex. For example, Alzheimer's disease, which is the most common neurodegenerative disease in old age (Ballard et al., 2011; Geldmacher, 2012), is associated with several genetic, nutritional, cognitive, and neural changes that amount to 100s of variables. Standard statistical methods as used in most empirical studies are ill-equipped to diagnose and understand AD. Big data methods will allow us to select the most important features that differentiate AD patients from healthy individuals, as this will allow clinicians and neurologists to only test these variables in clinical practice.

**Figure 1:**
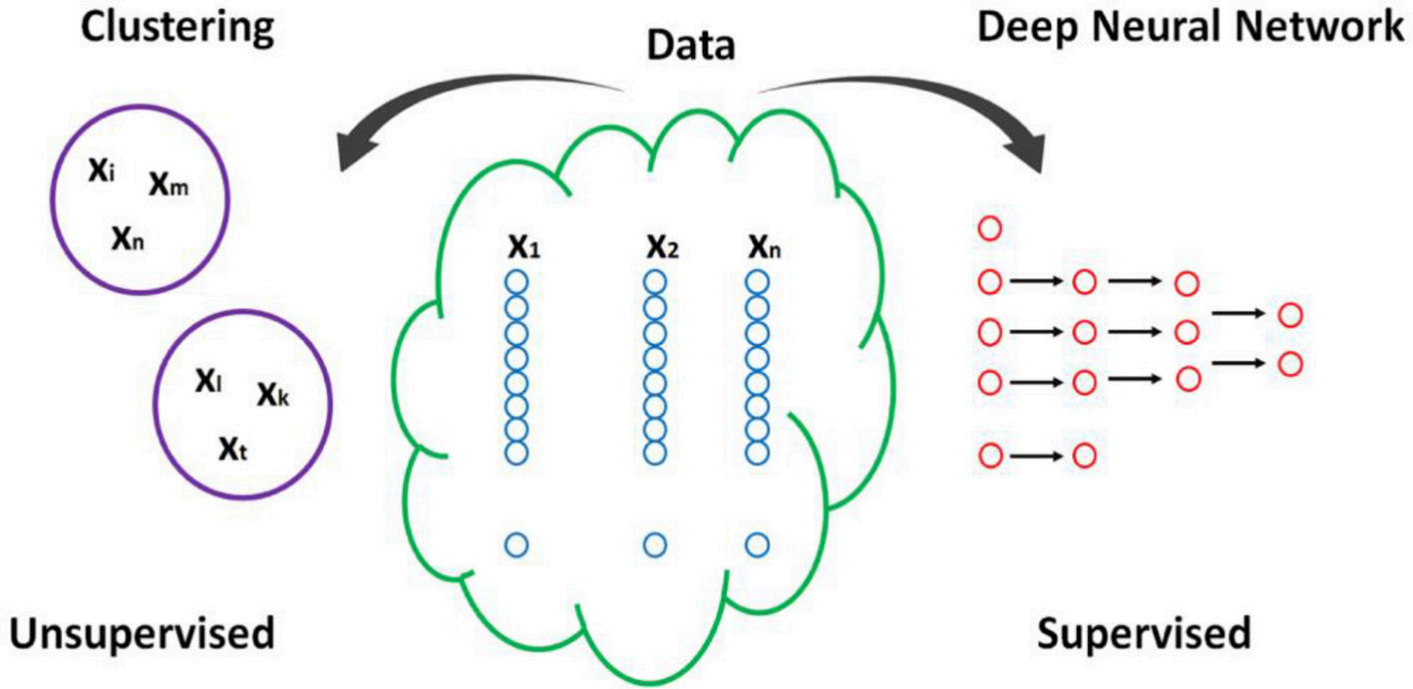
A schematic explaining the clustering and DNN proposed here. X_j_ refers to feature j, and Y_i_ refers to individual i. n here is numbers of features, and m to a number of individuals.

As human behavior is extremely complex, it is no surprise that many existing findings in the field of psychology and medicine are conflicting. This is perhaps due to the existence of several factors affecting human behavior as well as the simplicity of theories used to explain human behavior (which is due to theory-driven approach in the field, as we discussed above). However, most psychological experiments mostly focus on measuring 2–7 variables. Most standard statistical methods cannot handle datasets with a large number of variables. Further, many of the small datasets cannot answer questions about causality. To do so, researchers often needs to collect longitudinal and big datasets. Below, we describe how machine learning methods, such as clustering and deep learning, can be applied to big datasets to solve complex psychology problems (see Figure 1).

## The application of clustering methods in psychology

Clustering is the process of partitioning a set of individuals (or objects) into subgroups. Accordingly, a cluster is a collection of data points that are similar to one another and dissimilar to data points in other clusters (Escudero et al., 2011). Clustering methods seek to segment the entire dataset into relatively homogeneous subgroups or clusters, where the similarity of data points within the cluster is maximized, and the similarity to data points outside this cluster is minimized (Larose, 2005). The clustering problem is defined as follows: Given a set of points in the multidimensional space, find a partition of the points into clusters so that the points within each cluster are similar to one another. Traditional clustering describes clusters using measures of similarity, such as Euclidian distance, and considers data points belonging to one and only one cluster at the time.

However, one type of clustering method known as fuzzy clustering, allows data points to belong to several clusters at the same time but with different membership degrees (Ahmadi et al., 2018). Fuzzy clustering has many applications to health sciences, as some individuals may or may not be diagnosed with a certain disorder, depending on different conditions.

Many psychological studies often start with dividing participants into some clusters. For example, many psychology studies divide participants into patient or control, urban or rural, and so on. However, clustering methods allow us to cluster data based on similarities among members/elements. In doing so, clustering methods can divide data into several clusters, and not necessarily 2 only. For example, instead of human tendency to divide participants into patient or control, clustering algorithms can subgroup these participants into 2, 3 or more clusters, perhaps pointing to several subgroups of patients. It is also possible that the rural/urban data involve several clusters, as urban people may be subdivided into several subclusters. By using clustering methods, we may be able to find more important relationships among participants than often assumed a priori. In one recent study, Crouse et al. (2018) used a clustering method, known as Hierarchical Agglomerate, to subtype psychosis-prone individuals. Unlike standard clustering algorithms, this approach assumes each element has its own cluster and then clusters are merged based on similarity in a hierarchical manner. Results show that there are three clusters, which differ in IQ and social functioning. Future research should use similar methods to subtype participants instead of using a priori (assumed) taxonomy.

## The application of deep learning methods in psychology

Deep neural networks (DNNs) are commonly used to classify data in different fields (LeCun et al., 2015; Amoroso et al., 2018; Wang et al., 2018). DNNs are non-linear methods that allow the learning of complex patterns among features, thus providing a complex non-linear classification of input data (Graepel et al., 2010). With more hidden layers in the network, the data becomes more easily separable due to non-linear transformations along different layers of the network (Plis et al., 2014). Thus, DNNs are able to utilize different feature combinations and thus could potentially improve classification of complex datasets. This has several benefits over linear classification models that often ignore complex interactions among features. In recent years, DNNs have gained great importance, especially because they better manage raw data than classical machine learning algorithms, thus they do not require a strong effort by human experts to denote which variables should be considered and which not on order to detect significant patterns within the data. Besides, the availability of huge computational resources (thanks to cloud technology) allows an intensive use of deep learning algorithms. Importantly, DNNs can be used to help predict who may develop a certain disorder, which is very important for providing an effective treatment for the patients (Choi et al., 2018). DNNs can also be used to predict academic performance of students based on their input data. Importantly deep learning can be used to extract key features underlying category membership (known as feature selection).

### Feature extraction

Different machine learning methods, such as the random forest algorithm, allow researchers to find best features/variables to explain differences among two or groups of participants (Amoroso et al., 2018). There are several ways to conduct feature selection. For example, one study used weight pruning methods in the Input Layer to find relevant features (Roy et al., 2015). Similarly, Munsell et al. (2015) have used the elastic net algorithm to reduce features and network connections. Nezhad et al. (2017) also identified the most relevant features underlying the occurrence of hypertension using an auto-encoder network. Recently, Zhang et al. (2016) have used Discriminant Autoencoder Network with Sparsity Constraint (DANS) to extract most important features that discriminate schizophrenia patients from healthy individuals. They reported some larger weight value in the network for certain features (connectivity of some brain areas, including cortex, basal ganglia, and cerebellum) best differentiate between the two populations.

In contrast, in standard psychology studies, usually experimental scientists test differences usually between one or two variables. As an example, a standard psychology study may investigate differences in quality of life, depression, stress, and so on in urban vs., rural participants. The study will then investigate if each of these variables or perhaps an interaction among two (or more) of them is significantly different. Multivariate classification methods allow the researcher to unveil strategic roles played by a set of variables, weak if considered on their own and which therefore could be disregarded. However, deep learning methods can test the differences among all variables, which can be in the order of 100s. In one recent study, Guo et al. (2015) used deep learning methods to find factors underlying academic performance of students. The networks included background, school-related, past study performance, and personal data, among other variables. The network was able to find a subset of these variables that predict academic performance (network output). Accordingly, psychology researchers can benefit from these findings by focusing on improving scores on variables related to better academic performance. Similarly, selecting key features has clinical importance, as it helps provide neurologists and clinicians with most important features that classify the sample (e.g., patient vs. healthy individual). Based on feature selection algorithms, neurologists can then only focus on collecting and measuring data related to these features in future diagnostic work.

## Conclusions

We here argue that the more data we collect, the better our understanding of human behavior will be. Instead of relying on theory-driven methods as often the case in psychology studies, big data approaches can drive discovery and let new “theories” arise directly from data. In addition, big data methods can provide unexpected results on subtypes of participants as well as better understand the nature of human behavior.

## Author contributions

All authors listed have made a substantial, direct and intellectual contribution to the work, and approved it for publication.

### Conflict of interest statement

The authors declare that the research was conducted in the absence of any commercial or financial relationships that could be construed as a potential conflict of interest. The reviewer XZ and handling Editor declared their shared affiliation at the time of review.
